# Magnetometry for Agriculture and Animal Systems: From Classical Sensors to Quantum-Enabled Biosensing

**DOI:** 10.3390/bios16060316

**Published:** 2026-06-01

**Authors:** Zixuan Wang, Xiaoyu Zhang, Kexun Tang, Liming Wu, Yuxiang Huang, Ning Zhang, Bei Wang, Xiaolong Wang, Yi Ruan, Qiang Lin

**Affiliations:** 1 Key Laboratory of Quantum Precision Measurement of Zhejiang Province, College of Science, Zhejiang University of Technology, Hangzhou 310023, China; kexuntang@zjut.edu.cn (K.T.); 2111709317@zjut.edu.cn (L.W.); 121222090012@zjut.edu.cn (Y.H.); beiwang@zjut.edu.cn (B.W.); xlwang@zjut.edu.cn (X.W.); yiruan@zjut.edu.cn (Y.R.); 2 Research Center for Life-Sciences Computing, Zhejianglab, Hangzhou 311100, China; zhangxiaoyu@zhejianglab.org (X.Z.); ningzhang@zhejianglab.org (N.Z.); 3 State Key Laboratory of Ocean Sensing, Institute of Quantum Sensing, School of Physics, Zhejiang University, Hangzhou 310058, China

**Keywords:** agricultural magnetometry, quantum biosensing, precision agriculture, biomagnetism, digital twin

## Abstract

Magnetic sensors offer a physically grounded and non-invasive approach to probing biological processes that remain inaccessible to optical, electrochemical, and radio-frequency techniques in complex agricultural environments. In recent years, advances in both classical and quantum magnetic sensors have enabled the detection of bioelectromagnetic signals across plants, soils, animals, and aquatic systems, spanning spatial scales from ionic currents to organ-level electrophysiology and population-level dynamics, positioning magnetometry as an emerging modality within the broader biosensor landscape. This review surveys the evolution of magnetic sensing technologies for agricultural and animal systems, from robust classical sensors used in navigation and soil mapping to quantum-enabled platforms, including Optically Pumped Magnetometers (OPMs) and Nitrogen-Vacancy (NV) centers, capable of resolving pT to fT biomagnetic signals. We synthesize the characteristic amplitudes, frequency ranges, and physiological origins of agriculturally relevant magnetic signals, and critically assess how techniques originally developed for medical magnetoencephalography, magnetocardiography, and low-field magnetic resonance imaging (LF-MRI) are being translated into field-deployable agricultural applications. Beyond sensing hardware, we highlight the essential role of artificial intelligence in extracting weak biological signals from dominant environmental noise, enabling synthetic gradiometry, low-field image reconstruction, and scalable interpretation in unshielded settings. Finally, we discuss how the integration of magnetic biosensing with digital twins supports predictive, multiscale monitoring of plant, animal, and ecosystem health. Together, these developments position magnetometry as an enabling technology for next-generation biosensors in precision and sustainable agriculture.

## 1. Introduction

The agricultural sector is undergoing a fundamental transformation from a paradigm dominated by chemical inputs and mechanization to one defined by information density, biophysical monitoring, and predictive modeling. This transition—often termed Agriculture 4.0 or smart farming—reflects the recognition that sustainable productivity gains increasingly depend on high-resolution sensing of the physiological states of plants, animals, and their surrounding environments [1,2]. Within this framework, optical and electrochemical sensing technologies have led the first wave of precision agriculture, enabling non-destructive assessment of crop vigor, nutrient status, pathogen presence, and soil chemistry, and establishing data-driven management as a practical reality [3].

In the broader sensing landscape, these technologies are widely recognized as core components of modern biosensors, where biological processes are translated into measurable physical signals through optical, electrochemical, or mechanical transduction mechanisms. As agricultural monitoring increasingly targets physiological and biochemical dynamics in living systems, the boundaries between environmental sensing and biosensing are becoming progressively intertwined.

However, large-scale deployment has revealed intrinsic physical and operational constraints. Optical sensing is limited by photon propagation in highly scattering and absorbing media, with soil opacity, canopy occlusion, tissue heterogeneity, and environmental illumination variability limiting penetration depth and compromising signal robustness, particularly for subsurface roots and deep-tissue physiology [4,5,6]. Electrochemical sensors, while chemically specific, typically require direct contact with soil or biological fluids, making them vulnerable to biofouling, electrode degradation, calibration drift, and sensitivity to temperature, pH, ionic strength, and moisture fluctuations during long-term field operation [7,8,9]. Consequently, first-wave sensing modalities primarily probe surface-accessible or chemically mediated signals and offer limited access to the fast, weak, and spatially distributed electrophysiological processes underlying plant signaling, root–soil interactions, and animal organ function. These processes are often buried within opaque media and evolve on timescales that challenge conventional optical and electrochemical approaches, motivating the search for alternative physical observables.

Magnetism offers such an alternative. From a biosensing perspective, magnetic fields generated by biological activity can be interpreted as endogenous biological signals arising from ionic currents, electrophysiological processes, and biogeochemical reactions. In this sense, magnetic sensing provides a complementary transduction pathway capable of probing biological function in a label-free and non-contact manner. Unlike electric fields or optical signals, magnetic fields propagate through biological tissue, soil, and structural materials with minimal attenuation or distortion, enabling non-invasive access to deeply embedded physiological and environmental processes [10,11,12]. Historically, agricultural applications of magnetism have been constrained by a pronounced performance gap: classical sensors were well suited for macro-scale environmental mapping and autonomous navigation [13,14], whereas the micro-scale domain of endogenous biomagnetic activity remained largely beyond reach. Classical fluxgate and magnetoresistive sensors, while robust, lacked the sensitivity to detect the ultra-weak biomagnetic fields generated by living organisms, which typically range from sub-femtoteslas (fT, 10^−15^ T) to picoteslas (pT, 10^−12^ T) [11,15]. Conversely, Superconducting Quantum Interference Devices (SQUIDs), capable of detecting such fields, were tethered to laboratory settings by the requirement for cryogenic cooling [16,17].

Recent advances in quantum-enabled magnetometry have fundamentally altered this landscape. OPMs operating in the Spin-Exchange Relaxation-Free (SERF) regime and room-temperature NV diamond sensors now provide laboratory-grade sensitivity in compact, scalable, and field-deployable form factors [18,19,20], enabling the direct detection of plant action potentials (APs) [21,22], non-invasive monitoring of animal cardiac activity [10,23], and high-resolution mapping of soil magnetic properties without the logistical burden of cryogenics [24,25]. Beyond these specific demonstrations, breakthroughs in programmable quantum sensing—achieving near-optimal metrology through entanglement and on-device learning [26,27]—underscore a broader transition of magnetometry from a geophysical and biomedical niche to a versatile, field-ready biosensing and bio-monitoring modality.

Together, these advances define a second, more fundamental wave of agricultural sensing, driven by the convergence of magnetometry, quantum physics, and data-centric modeling. In this emerging paradigm, magnetic fields act not merely as environmental descriptors but as direct, non-invasive reporters of biological function. In this context, biomagnetic signals can be regarded as intrinsic biological markers, positioning magnetic sensing as an emerging label-free biosensing strategy within precision agriculture.

Accordingly, magnetic sensing in agriculture can be viewed as a rapidly emerging branch of magnetic biosensing, where advances in sensor physics, quantum measurement, and data analytics converge with biological monitoring needs. This report provides a critical analysis of this technological convergence. It dissects the physical principles governing sensor evolution, from the saturation mechanics of fluxgates to the quantum spin dynamics of alkali vapors. It characterizes the biological sources of magnetic fields in agriculture, defining the ionic mechanisms of plant signaling and the electrophysiology of ruminant cardiology. Furthermore, it evaluates the diverse applications of these technologies across three critical domains: the phenotyping of plant systems, the health monitoring of terrestrial livestock, and the navigation and containment of aquaculture species. Finally, the report examines the integration of these complex data streams through Artificial Intelligence (AI) and the emergence of Biomagnetic Digital Twins (BDTs), proposing a future where magnetic biosensing serves as a foundational layer of the precision agriculture ecosystem.

## 2. Foundations of Magnetic Biosensing in Agriculture: Transduction Mechanisms, and Sensor Evolution

Magnetic sensing has evolved from a tool for geophysical exploration into an emerging modality for biosensing and biological monitoring. In agricultural and livestock systems, magnetic measurements can provide both environmental context and biologically specific information, ranging from soil magnetization to electrophysiological activity and magnetic nanoparticle-based bioassays. Early instruments such as fluxgate magnetometers in the 1930s offered nanotesla (nT, 10^−9^ T) sensitivity suitable for geomagnetic surveys [28], but the progressive improvement of sensitivity—from nT to fT levels—has gradually opened the possibility of detecting biologically relevant magnetic signatures and magnetically labeled biomolecules [11,29,30,31,32].

**Figure 1:**
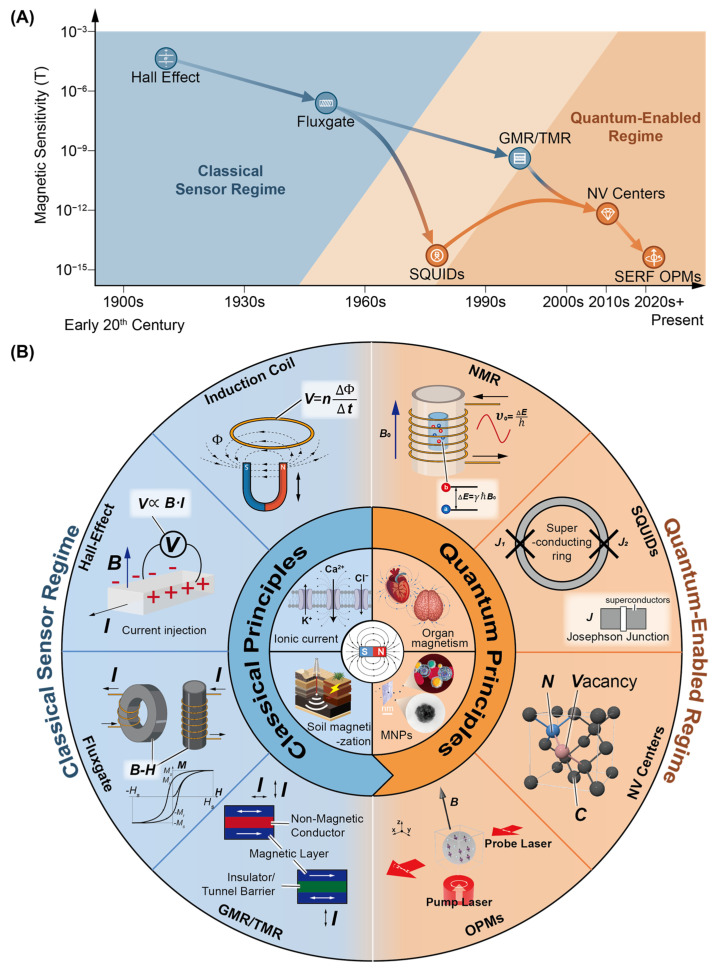
Schematic of the spatiotemporal evolution and physical mechanisms of magnetic sensing architectures: from classical transduction to quantum-enabled detection. (**A**) Evolutionary trajectory and sensitivity paradigm shift. The classical sensor regime (blue zone) relies on solid-state carrier transport and magnetic flux modulation, whereas the quantum-enabled regime (orange zone) denotes detection platforms governed by wavefunction coherence. (**B**) Mechanistic framework of biomagnetic detection. A concentric mapping connects magnetic signal origins with agricultural applications. The inner ring includes ionic currents, organ magnetism, soil magnetization, and magnetic nanoparticles (MNPs). The outer ring maps corresponding transduction physics across sensor classes, spanning classical Faraday induction to quantum-mechanical detection mechanisms. The inclusion of magnetic nanoparticles and biochemical binding events in the inner ring highlights the interface between physical transduction mechanisms and biological recognition, forming the foundation of hybrid magnetic biosensor architectures.

Magnetic sensing technologies are increasingly integrated with biological recognition elements to form hybrid magnetic biosensors capable of selective biochemical detection. The effectiveness of magnetometry in agriculture ultimately depends on matching sensor performance—sensitivity, bandwidth, and dynamic range—to the target biological or environmental signal. As summarized in Figure 1, the historical transition from classical to quantum sensors reflects a continuous effort to bridge the sensitivity gap between environmental magnetism and endogenous biomagnetic activity, and magnetic nanoparticle-based biochemical detection, thereby establishing the transduction foundations of hybrid magnetic biosensors.

### 2.1. Magnetic Sensors Based on Classical Principles

This section covers sensors whose operation is fundamentally governed by classical electromagnetism and electron transport theories. While modern implementations, particularly at the nanoscale, may involve quantum effects such as tunneling, their macroscopic readout (e.g., voltage or resistance) distinguishes them from the next generation of quantum sensors that manipulate atomic coherence states. Classical magnetometers remain the industry standard for agricultural environmental mapping and machine guidance due to their maturity, robustness, and cost-effectiveness. Within the biosensor ecosystem, these platforms primarily provide environmental context, magnetic labeling readout, and infrastructure monitoring that complement biochemical sensing technologies.

Fluxgate magnetometers operate via the magnetic saturation of a ferromagnetic core, where external fields generate measurable voltage harmonics [33]. Offering high sensitivity (10–100 pT/Hz^1/2^) and wide temperature ranges (−55 °C to +175 °C) [33,34], they excel in harsh agricultural conditions for soil mapping and ferrous anomaly detection [35]. However, their large footprint and power consumption (tens of mW) limit deployment in ultra-compact IoT livestock monitors [36]. These capabilities make fluxgates valuable for large-scale environmental characterization that supports biosensing deployments.

Hall-effect sensors are mature, CMOS-integrated microsystems operating in the mT regime [37,38]. Their stability and offset reduction are enhanced by geometric optimization, current spinning, and data-driven designs [39,40], while wide-bandgap structures (e.g., AlGaN/GaN) improve high-temperature operation [41]. Often benchmarked alongside MR technologies [42], they are robust against dust and vibration, making them ideal for non-contact machinery measurements (e.g., wheel speed, current) [43]. Thermal drift and field attenuation are typically mitigated using dual-sensor and field-shaping strategies [44]. In biosensing infrastructures, Hall sensors frequently serve as auxiliary detectors for actuator feedback, magnetic nanoparticle tracking, and embedded instrumentation.

MR sensors transduce magnetic fields into resistance changes through spin-dependent transport, comprising three generations: AMR (angle-dependent), GMR (spin-dependent scattering across conductive spacers) [45], and TMR (spin-dependent quantum tunneling across insulating barriers) [36]. Despite its quantum tunneling mechanism, TMR serves as a bridge to quantum sensing due to its classical macroscopic readout [46]. With ~1 nT sensitivity and ultra-low power consumption (~μW), MR sensors support wireless networks, ingestible rumen boluses [45] and dense tracking arrays [47]. Ultimately, AMR, GMR, and TMR are cornerstones of agricultural IoT systems [48], providing low-power magnetic readout for distributed biosensing nodes while remaining fundamentally distinct from true atomic quantum sensors.

MR sensors functionalized with antibodies or nucleic-acid probes enable highly sensitive detection of proteins, pathogens, and nucleic acids using magnetic nanoparticle labels. A comprehensive review by Wu et al. highlighted the rapid development of GMR biosensors in biomedical applications, emphasizing their compatibility with microfluidics, CMOS fabrication, and multiplexed detection [49]. Earlier, Gaster et al. demonstrated matrix-insensitive protein assays using GMR sensors, achieving highly sensitive detection of cancer biomarkers directly in complex biological fluids such as serum [50], thereby establishing magnetic immunoassays as a powerful alternative to optical biosensors. These advances demonstrate that classical magnetic sensors are already deeply integrated into hybrid biosensor platforms that combine magnetic transduction with biological recognition elements such as antibodies, aptamers, and DNA probes.

However, while magnetic nanoparticle labeling enables highly selective biochemical detection, the direct measurement of endogenous biomagnetic activity remains beyond the sensitivity limits of classical devices, motivating the development of quantum-enabled biosensing architectures discussed in the following section.

### 2.2. Quantum-Enabled Sensors

While classical sensors remain essential for environmental mapping and machine guidance, their sensitivity limits prevent detection of endogenous biomagnetic fields. In precision agriculture, an emerging frontier is the non-invasive monitoring of electrophysiological processes such as plant action potentials and livestock magnetocardiography, whose magnetic signatures lie in the pT–fT range and remain below the noise floor of conventional fluxgate and TMR devices.

Bridging this gap requires coherent quantum sensors—including SQUIDs, OPMs, and NV centers—which exploit superconducting interference or long-lived spin coherence to operate near fundamental quantum noise limits. Beyond passive biomagnetic monitoring, these platforms are increasingly integrated with magnetic nanoparticles, antibodies, and molecular probes, enabling hybrid magnetic biosensors capable of selective biochemical and cellular detection. Together, these technologies transform agricultural magnetometry from environmental measurement into a biosensing and diagnostic modality for real-time stress phenotyping and health monitoring.

SQUID magnetometers represent the historical sensitivity benchmark of biomagnetism, operating through superconducting quantum interference to convert minute magnetic flux changes into measurable voltage signals [51,52,53,54]. Low- Tc devices achieve sub-fT sensitivity, while high-Tc systems offer improved practicality at the expense of higher noise [55,56,57]. Recent developments in cryogen-free cooling have reduced system complexity [58,59]. However, the requirement for cryogenic operation imposes logistical constraints and increases sensor–source distance, limiting field deployment. Consequently, in agricultural biosensing, SQUIDs primarily serve as laboratory reference instruments used to validate ultra-weak plant and animal biomagnetic signals before translation to room-temperature platforms [31,60,61,62].

Atomic magnetometers and SQUIDs have also been integrated with magnetic nanoparticle-based bioassays for selective biomolecule detection. Johnson et al. demonstrated magnetic relaxometry using both atomic magnetometers and SQUID sensors to detect targeted cancer cells labeled with antibody-functionalized magnetic nanoparticles [63]. This work established the feasibility of combining ultra-sensitive magnetometry with biological recognition elements to enable highly specific cellular detection, illustrating how quantum magnetometry can operate as the transduction backbone of hybrid magnetic biosensors for highly specific cellular and molecular diagnostics.

Room-temperature OPMs, particularly in the SERF regime, have emerged as the most promising platform for agricultural biomagnetic sensing. These sensors exploit optically polarized alkali-vapor spin ensembles operating in near-zero magnetic fields to suppress spin-exchange relaxation, enabling fT-level sensitivity comparable to SQUIDs without cryogenics [18,21,32,61,64,65,66]. Their compact, thermally insulated architectures enable wearable and conformable sensor arrays for plants and livestock. Field-deployable systems and commercial instruments now support applications ranging from plant action potential detection to livestock magnetocardiography, enabling minimally invasive, longitudinal monitoring of physiological function [22,67,68,69]. To further enhance OPM performance beyond spin-exchange–limited sensitivity, weak-measurement–assisted optical readout has emerged as a promising strategy for mitigating technical noise and improving detection contrast under realistic experimental conditions [70,71,72].

Importantly, weak measurement has already demonstrated clear value in biosensing. In practical sensing contexts, quantum weak measurement enhanced protein-induced optical shift detection in a molecular imprinting platform [73], weak-value amplification improved enantiomer discrimination under non-ideal conditions in chiral sensing [74], and a common-path implementation enabled stable, label-free biomolecule detection through suppression of environmental phase noise [75]. These demonstrations highlight its capability to amplify subtle optical signatures associated with biochemical interactions.

More recently, biased weak measurement schemes were theoretically investigated for enhancing Fisher information and magnetic sensitivity in SERF magnetometers [76], while inverse weak measurement protocols were reported to improve robustness against laser power fluctuations and long-term drift in SERF configurations [77]. Collectively, these advances position weak-measurement-assisted readout as a key pathway for translating high-sensitivity OPMs into noise-intensive agricultural biosensing environments.

NV centers in diamond provide a complementary quantum sensing modality with nanoscale spatial resolution. Through optically detected magnetic resonance, the Zeeman shift in NV spin states enables room-temperature magnetic field sensing at nanometer-to-micrometer scales [19,78,79,80]. Their chemical inertness, photostability, and biocompatibility enable intracellular and micro-scale sensing without the limitations of conventional fluorescent probes [81,82,83,84]. In agriculture, NV sensors are particularly suited to cellular-level investigations, including ion transport, magnetic tracer tracking, and nutrient uptake studies. This capability complements OPM-based organ-level monitoring by enabling investigation of the cellular mechanisms underlying crop stress and plant physiology [85,86].

Beyond physiological sensing, NV-based magnetometry has recently enabled digital magnetic detection of biomolecular interactions at the single-nanoparticle level. Chen et al. demonstrated magnetic detection of individual magnetic nanoparticles bound to biomolecules, achieving digital counting of molecular binding events and illustrating the potential of NV centers for single-molecule–level biosensing [87]. More recently, quantum sensing with NV centers enabled in situ imaging of free radicals during chemical reactions, providing direct access to transient biochemical processes under realistic conditions [88]. These advances highlight the rapid transition of NV magnetometry from a physical sensing technology to a powerful platform for selective chemical and biochemical biosensing.

### 2.3. Comparative Analysis and Selection Criteria

**Figure 2:**
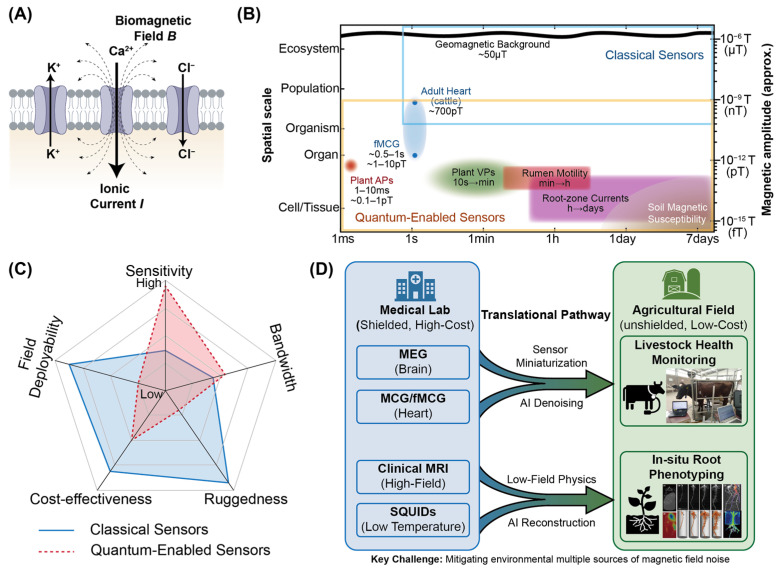
The landscape of agricultural magnetometry: From biological signal origins to field-deployable translational pathways. (**A**) Biophysical origin of biomagnetic fields. The fundamental mechanism of bioelectromagnetism in agricultural systems is driven by membrane depolarization and subsequent ion transport (e.g., K^+^, Cl^−^). These trans-membrane ionic currents generate accompanying magnetic fields according to the Biot–Savart law, providing a non-invasive window into physiological states. (**B**) Spatiotemporal distribution and detection limits. Agricultural magnetic signatures are mapped across spatial (cell/tissue to ecosystem) and temporal (1 ms to 7 days) scales. The vertical axis illustrates a formidable detection challenge: ultra-weak biological signals, such as plant action potentials (~0.1–1 pT), reside roughly nine orders of magnitude below the dominant geomagnetic background (~50 µT). The bounding boxes distinguish the operational limits of classical sensors (blue) and quantum-enabled platforms (orange), highlighting the necessity of quantum technologies for resolving transient, ultra-low-field biomagnetic phenomena. (**C**) Multi-dimensional performance trade-offs. A radar chart comparing classical (blue solid line, e.g., Fluxgate, Hall) and quantum-enabled (red dashed line, e.g., SERF OPM, NV centers) sensing architectures across five critical dimensions: sensitivity, bandwidth, ruggedness, cost-effectiveness, and field deployability (high is better). (**D**) Translational framework for precision agriculture. The roadmap for migrating high-precision magnetometry from shielded, high-cost medical laboratories (e.g., MEG, SQUIDs) to unshielded, low-cost agricultural environments. This transition is critical for enabling real-world applications, such as proactive livestock health monitoring and in situ root phenotyping. Reproduced from Ref. [69] under the CC BY 4.0 license. © 2020 The Authors. Published by Elsevier Ltd. Reproduced from Ref. [90] under CC BY-NC-ND 4.0 license. © 2018 The Authors. Published by Elsevier Ltd.

The selection of a magnetometer for agricultural deployment involves a multi-dimensional optimization of sensitivity, bandwidth, power consumption, ruggedness, and cost. As synthesized in Table 1, a distinct bifurcation exists in the current technology landscape. On one side of the spectrum, classical sensors (Fluxgate, Hall-effect) offer the mechanical robustness and high dynamic range necessary for macro-scale tasks. For instance, autonomous navigation in unstructured terrain or mapping soil drainage infrastructure often favors these sensors due to their low cost and vectoral stability under motion. However, as Figure 2A,B shows, their fundamental physical limitation lies in the detection of fT-scale fields characteristic of biological signaling.

Conversely, quantum-enabled sensors (SQUIDs, OPMs) overcome this sensitivity barrier, enabling the micro-scale interrogation of plant electrophysiology and animal cardiology. However, this quantum leap in sensitivity introduces complex trade-offs regarding measurement modality, operational cost, and environmental isolation. While scalar OPMs mitigate heading errors for drone-based soil surveys, the vectoral SERF OPMs essential for biomagnetic source localization necessitate strict null-field environments and are highly sensitive to orientation. Beyond these physical constraints, the prohibitive size, weight, power, and cost metrics of current quantum systems restrict their deployment density compared to ubiquitous solid-state arrays, as synthesized in Figure 2C.

It should be emphasized that the field performance of SERF OPMs on genuinely moving agricultural platforms remains highly constrained. SERF operation requires the residual magnetic field to be reduced to near-zero levels, and the sensors typically exhibit a limited dynamic range and strong sensitivity to field gradients, sensor orientation, vibration, and platform motion. Although active compensation coils can enable operation outside conventional magnetically shielded rooms, their effectiveness is greatest in stationary or quasi-stationary settings where the background field and sensor geometry remain relatively stable. For this reason, OPMs should currently be regarded as more suitable for controlled barns, restrained-animal measurements, portable shielded stations, or targeted operation in magnetically quiet zones than for fully mobile platforms such as drones or free-ranging livestock [69] (Figure 2D). The near-zero-field requirement, and compensation architecture associated with SERF OPMs operation are discussed in detail in Section 3.6.

Beyond magnetic-field constraints, practical deployment is limited by system-level factors including cost, power consumption, thermal management, calibration burden, and operator expertise. SERF OPM systems require laser sources, vapor-cell heating, magnetic-field stabilization, and often multi-axis compensation coils, which increase size, weight, power consumption, and maintenance complexity. Long-term agricultural monitoring further requires stable calibration under temperature and humidity variations, protection from dust and mechanical shock, and robust procedures for sensor alignment and baseline drift correction. These requirements contrast with fluxgate, Hall-effect, and MR sensors, which are less sensitive but cheaper, more rugged, and easier to deploy in dense distributed networks. Thus, the near-term role of quantum magnetometers in agriculture is likely to be selective high-fidelity monitoring rather than ubiquitous field-scale sensing.

To place these sensor capabilities in the context of biological relevance, Table 2 summarizes the typical amplitudes and frequency ranges of biomagnetic signals encountered in agricultural and animal systems, benchmarked against the geomagnetic background. This quantitative contrast highlights why micro-scale physiological signals remain fundamentally inaccessible to classical sensors under field conditions, and why environmental noise suppression is a defining system-level challenge rather than a secondary consideration.

Crucially, the deployment of these sensors is further bifurcated by the strategy for environmental noise suppression, which generally follows two distinct pathways. The first approach employs active magnetic compensation using coil systems, enabling operation in unshielded open fields at the cost of increased architectural complexity and power consumption [91]. The second approach relies on passive low-noise magnetic shielding, which provides the extreme isolation required for limit-pushing sensitivity but confines measurements to laboratory-like conditions [65]. Therefore, the future of agricultural magnetometry lies not in replacing classical sensors with quantum ones, but in a hierarchical deployment strategy. This involves utilizing robust classical arrays for environmental characterization to facilitate the targeted operation of high-fidelity quantum sensors within magnetically quiet zones.

## 3. Magnetic Sensing in Bio-Agricultural Systems: From Mechanisms to Field Applications

While recent advances in quantum technology have pushed magnetic sensitivity into the sub-fT regime, magnetic biosensing in agriculture is ultimately constrained not by sensor performance alone, but by the nature of the biological magnetic sources it seeks to resolve. Across plant, animal, and soil systems, living processes generate electrical currents through ionic transport, membrane depolarization, and collective electrophysiological coordination. According to Maxwell’s equations, these currents are inevitably accompanied by magnetic fields whose amplitudes, spatial distributions, and temporal dynamics encode fundamental physiological information. Unlike optical or electrochemical observables, these biomagnetic signals propagate through opaque media—soil, tissue, and structural materials—with minimal distortion, offering a unique, non-invasive window into deeply embedded biological function. Consequently, the agricultural relevance of magnetometry is best understood not from the perspective of sensor technology alone, but through a signal-centric framework that links biological origin, magnetic observability, and actionable agronomic insight.

**Figure 3:**
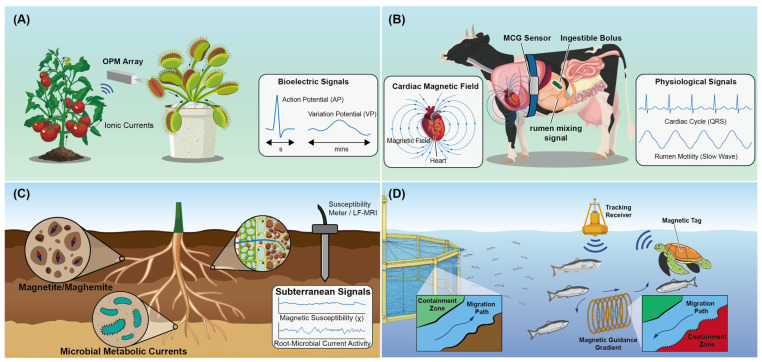
Cross-ecosystem application landscape of magnetic biosensing. Magnetic sensors provide a non-invasive, physically grounded window into biological processes across diverse environments. (**A**) Plant Systems: Detection of rapid systemic signaling, such as APs and VPs, using OPM arrays to monitor real-time stress responses without electrode-induced damage. (**B**) Terrestrial Livestock: Monitoring of cardiac health and digestive dynamics; magnetic fields bypass the electrical insulation of vernix and fur, enabling fMCG for fetal viability and tracking of ingestible magnetic boluses for rumen motility. (**C**) Soil and Root Zone: Assessment of rhizosphere health and microbial metabolic activity through LF-MRI and magnetic susceptibility (χ), resolving root architecture and nutrient-driven ionic currents in situ. (**D**) Aquaculture: Utilizing magnetic tagging and guidance gradients for individual tracking and behavioral control in optically opaque aquatic environments.

Within agricultural ecosystems, biomagnetic signals span an extraordinary range of spatial and temporal scales. In plants, fast APs, slow variation potentials (VPs), and circadian ionic rhythms generate magnetic signatures from the sub-pT to pT range, reflecting acute stress responses and long-term physiological adaptation. In livestock, coordinated cardiac, neural, and gastrointestinal electrophysiology produces stronger yet spatially complex magnetic fields that encode health, welfare, and developmental status, including fetal viability. At the soil–plant–microbial interface, magnetic phenomena emerge both from pedogenic mineral transformations and from microbial metabolic currents, forming an electromagnetic continuum that links soil chemistry, microbial ecology, and root function. By explicitly connecting magnetic signal characteristics to physiological processes and agronomic decision-making, we establish the physical foundation upon which sensor deployment strategies, AI–assisted signal extraction, and BDTs are constructed in subsequent sections. The multifaceted nature of these applications across plant, livestock, and soil environments is synthesized in Figure 3, which illustrates how diverse biomagnetic origins are translated into specific agricultural monitoring capabilities.

### 3.1. Plant Systems: From Ionic Currents to Stress Phenotyping

Plant systems represent the most information-dense yet least accessible domain of agricultural physiology. Unlike animal tissues, plant signaling is distributed, slow, and deeply embedded within optically opaque and mechanically heterogeneous structures. Electrical coordination in plants arises from ionic fluxes (e.g., Cl^−^, Ca^2+^, and K^+^) across vast cellular networks, spanning leaves, stems, and roots, and governs both acute stress perception and long-term adaptive responses. These ionic currents generate ultra-weak but structured magnetic fields that encode the timing and systemic reach of plant signaling events.

Organizing plant magnetometry by signal class, rather than by sensor modality, provides a unified framework for linking electrophysiological dynamics with stress phenotyping and resource management in precision agriculture. If crop plants generate magnetic signatures analogous to those observed in excitable species—albeit with weaker amplitudes—arrays of OPMs could function as early-warning systems in controlled environments such as greenhouses or vertical farms. As illustrated in Figure 3A, magnetic sensing captures the rapid propagation of APs and VPs in plants, providing a non-invasive alternative to traditional electrodes for real-time stress phenotyping. By detecting systemic electrical signaling traffic, including APs and VPs, prior to visible physiological damage, such platforms could trigger automated irrigation, climate control, or mitigation strategies in real time [21]. This signal-centric perspective establishes the conceptual foundation for the plant-focused magnetometric studies discussed below.

#### 3.1.1. Fast Electrophysiological Signals (APs)

Fast electrophysiological signaling in plants is mediated by APs, which enable rapid, long-distance communication in response to acute environmental perturbations. Although plants lack a centralized nervous system, excitable species exhibit well-defined APs that propagate along vascular tissues through coordinated ionic depolarization and repolarization [92]. These signals arise from synchronized transmembrane ion fluxes, typically involving chloride efflux and calcium influx during depolarization, followed by potassium-mediated repolarization, which generate transient axial ionic currents along conductive tissues [22]. These axial ionic currents generate weak but detectable magnetic fields that mirror the timing and shape of the underlying electrical signals. In excitable plant species, APs serve as rapid systemic signals in response to wounding, thermal shock, herbivory, and abrupt osmotic stress, initiating downstream physiological defenses [93]. Their fast, current-based nature renders them suitable candidates for non-invasive magnetic detection.

The biomagnetic signatures associated with plant APs are ultra-weak yet structured, with peak amplitudes typically in the sub-pT to pT range and dominant spectral content below 10 Hz. While APs are most prominent in rapidly excitable species such as *Dionaea muscipula* and *Mimosa pudica*, similar transient electrical signals have been observed in agronomically relevant crops including tomato (*Solanum lycopersicum*) and tobacco (*Nicotiana tabacum*) under acute stress conditions [22]. More recently, SERF OPMs have enabled non-contact detection of action potential-associated magnetic transients in *D. muscipula*, resolving heat-induced signals with peak-to-peak amplitudes of 0.5–1.0 pT through gradiometric noise suppression [94].

Beyond fundamental detection, the magnetic monitoring of APs provides a robust framework for evaluating plant responses to external stimuli without the physiological artifacts or tissue damage associated with electrode implantation into vascular tissues [22]. This non-invasive advantage is particularly significant in the context of stress physiology, where mechanical wounding from electrodes can itself trigger confounding electrical and biochemical responses. Although AP-associated magnetic signals in major row crops are expected to be weaker than those observed in highly excitable species, the technical capability to resolve these fast dynamics enables real-time identification of acute stress events. Electrophysiological studies have demonstrated that such rapid signals play an integral role in physiological adaptation under environmental perturbation [95], acting as intercellular messengers that coordinate downstream defense responses, including the synthesis of secondary metabolites [96]. Consequently, capturing this immediate systemic signaling via magnetic sensing offers transformative potential for precision agriculture by identifying stressors before visible symptoms manifest.

#### 3.1.2. Systemic Slow Waves (VPs and Hydraulic–Electrical Coupling)

Beyond fast excitation, plants rely predominantly on slower, decremental electrical signals, commonly termed VPs, to coordinate whole-organism responses to localized stress [97]. While fast APs occur in highly excitable species, most agricultural crops, including wheat (*Triticum aestivum*), maize (*Zea mays*), and tomato (*Solanum lycopersicum*), rely predominantly on slower, decremental electrical signals known as variation potentials (VPs) to coordinate systemic responses to localized stresses such as wounding or herbivory [98]. Unlike self-propagating APs, VPs are hydraulic–electrical waves: a local perturbation (e.g., tissue damage) triggers a transient change in xylem/phloem pressure, which activates mechanosensitive ion channels and drives coordinated ion fluxes across vascular membranes [99].

The ultra-low frequency of VPs (near-DC to 10 Hz) requires sensing platforms with exceptional baseline stability to mitigate 1/*f* noise [100]. Yet their successful resolution would provide a non-invasive, pre-symptomatic marker of physiological disruption [101], directly linking localized injury to whole-plant outcomes such as defense activation or growth suppression [102]. The relevance of magnetic sensing for plant physiology is further underscored by techniques like NMR, which exploits magnetic interactions to non-invasively image water distribution and transport dynamics in living plants [103]—demonstrating that magnetic methods can indeed capture key aspects of plant systemic function, albeit through different physical principles than biomagnetic field detection.

Beyond transient slow waves, persistent low-frequency ionic and hydraulic processes, driven by diurnal transpiration, phloem transport, and chronic osmotic stress, generate quasi-static biomagnetic signatures that reflect plant water status and metabolic health, offering early indicators of drought or salinity stress before visible symptoms appear [92,102]. Resolving these long-term dynamics enables precision irrigation scheduling and continuous plant health diagnostics, supporting optimized resource allocation in saline or water-limited agricultural environments.

#### 3.1.3. Root and Rhizosphere Electrodynamics

The root–soil interface is among the most electromagnetically active yet inaccessible regions of the agricultural ecosystem. Nutrient uptake, proton extrusion, and ion exchange across root membranes generate localized ionic currents within the rhizosphere, which have been shown to play a determinative role in root morphogenesis and growth polarity [104]. These currents produce magnetic fields that encode subterranean physiological activity, making magnetometry a unique, non-destructive tool for probing belowground function without disturbing soil or roots.

At the electrochemical level, the rhizosphere is a site of intense ionic activity driven by active nutrient acquisition and metabolic regulation [105]. The uptake of nitrate (${NO}_{3}^{-}$) and ammonium (${NH}_{4}^{+}$), coupled with proton ($H^{+}$) extrusion mediated by plasma membrane $H^{+}$-ATPase pumps, establishes localized current loops at this boundary. The resulting biomagnetic fluctuations, which can be linked to the magnetohydrodynamic effects of ionic currents in solution [106], reflect root metabolic vigor and nutrient acquisition efficiency. This continuous biomagnetic footprint facilitates root health assessment and early detection of subterranean stressors like nutrient imbalance, aluminum toxicity, and pathogens.

This localized electrodynamic activity is intrinsically linked to nutrient acquisition efficiency; magnetic treatment has been observed to enhance the uptake of both macronutrients (N, P, K) and micronutrients (Fe, Mn, Cu) in the rhizosphere [107], directly influencing the accumulation of proteins and soluble sugars within the plant matrix [108].

A key advantage of magnetic sensing is its ability to penetrate soil with negligible attenuation, overcoming the optical opacity and electrical impedance that limit traditional root imaging [90]. Continuous monitoring thus allows real-time evaluation of nutrient flux and uptake efficiency for root phenotyping under field conditions. Complementing this approach, LF-MRI provides a structural framework by resolving root architecture in natural soil. By leveraging the distinct differences in spin-spin relaxation times (*T*_2_) between bulk soil water and biologically constrained root water, this technique successfully isolates root geometries from complex soil backgrounds without physical excavation [109]. This mapping links magnetic signals to nutrient uptake, water transport, and stress adaptation.

Together, root and rhizosphere electrodynamics represent the slowest and most spatially embedded signal class in plant magnetometry. Integrated with fast excitation and systemic slow-wave signaling, these signatures complete a multiscale framework for continuous surveillance of belowground processes governing crop productivity and resilience.

#### 3.1.4. Core Mechanisms of Magnetic Field Interactions in Plant Systems

The integration of magnetometry into agricultural ecosystems requires a fundamental understanding of how magnetic fields interact with plant physiology. These interactions occur across multiple spatial and temporal scales, encompassing quantum, biochemical, and systemic responses [105]. At the physiological level, magnetic fields have been shown to significantly improve photosynthetic parameters, including maximum quantum efficiency, electron transport quantum yield, and overall photosynthetic rate [110]. This enhanced photochemical performance directly increases light absorption efficiency, driving greater biomass accumulation and heightened plant vigor [111].

Beyond primary metabolism, magnetic field interactions extend to cellular signaling and stress modulation. Static magnetic field treatment typically accelerates the formation and accumulation of reactive oxygen species (ROS) within plant tissues [112]. As a systemic response, plants simultaneously induce the high-level expression of antioxidant enzymes, such as superoxide dismutase, peroxidase, and catalase, which effectively mitigates oxidative stress damage and maintains redox homeostasis [105,113]. Furthermore, moderate-intensity static magnetic fields significantly enhance membrane permeability by altering plasma membrane structure, thereby modulating ion activation states and the dynamics of downstream metabolic pathways [107].

At the molecular and quantum levels, magnetic fields act as environmental signals that activate cellular stress response mechanisms and regulate gene expression associated with biosynthesis and cellular adaptation [114]. For instance, magnetic intervention upregulates antioxidant genes and iron transporter genes, reinforcing the plant’s metabolic resilience [115]. These diverse biological effects are anchored in fundamental physical processes, most notably the radical-pair mechanism. This theory posits that magnetic fields alter the kinetics of enzyme-catalyzed reactions by influencing the singlet-triplet interconversion of radical pairs during biochemical transitions [116]. Complementary models, such as ion cyclotron resonance and the interference of magnetic fields with the dynamic transport of paramagnetic ions (e.g., manganese) within chloroplasts, provide additional frameworks for understanding the complex bio-magnetic coupling in plant systems [117]. The engineered MagLOV2 protein exemplifies this mechanism by achieving a 10-fold amplification of magnetic field effects in fluorescence modulation, demonstrating its utility as a quantum sensor for magnetic field detection in biological contexts [118].

### 3.2. Terrestrial Livestock: Cardiac, Neural, and Gastrointestinal Magnetism

In animal systems, biomagnetic signals arise from highly organized, excitable tissues whose coordinated electrical activity governs health, behavior, and development. Cryogenic SQUIDs provided the first experimental confirmation that these electrophysiological events are accompanied by detectable magnetic fields [56]. Cardiac contraction, neural processing, and gastrointestinal motility generate magnetic fields that are orders of magnitude stronger than those observed in plants, yet spatially complex and embedded within heterogeneous tissues. The advantage of magnetism in bypassing insulating barriers is central to livestock health monitoring (Figure 3B), where fMCG can resolve fetal cardiac cycles through tissue and vernix that typically attenuate electrical signals. Unlike surface electrodes, magnetic sensing captures these processes non-invasively and without distortion from insulating layers or motion-induced artifacts. Organizing livestock magnetometry by physiological subsystem reveals how magnetic observables can serve as objective indicators of welfare, metabolic stability, and reproductive success.

#### 3.2.1. Cardiac Dynamics and Fetal Viability

The mammalian heart is the most potent source of endogenous biomagnetic fields in terrestrial animals. In adult cattle, rhythmic ventricular depolarization and repolarization create current dipoles that generate peak magnetic fields of 10–100 pT at the thoracic surface [62]. However, the most transformative application of veterinary MCG lies in fMCG. fMCG.

In pregnant livestock, traditional fetal electrocardiography (fECG) often fails because the vernix caseosa—a waxy, insulating layer on the fetal skin—effectively short-circuits electrical signals, masking them from surface electrodes. Magnetic fields, by contrast, completely permeate this insulating barrier and maternal tissues without attenuation. This unique physical advantage enables fMCG to detect the fetal QRS complex with high signal-to-noise ratio, allowing for the precise calculation of fetal heart rate variability (fHRV) [52]. Reduced fHRV serves as a precise physiological marker for hypoxia, distress, or congenital defects, facilitating proactive veterinary intervention in high-value breeding programs. Research has demonstrated that tracking fHRV trajectories serves as a highly precise marker for the development of the autonomic nervous system [62]. The high cost of using SQUIDs is one of the limiting factors for fMCG, and the development of OPMs and shieldings has made fMCG more practical [119]. Consequently, fMCG provides an unparalleled, non-invasive window to diagnose fetal arrhythmia, hypoxia, or distress, facilitating proactive veterinary interventions to save offspring in high-value breeding programs [120].

#### 3.2.2. Neural Activity and Objective Welfare Assessment

Neural electrophysiology generates ultra-weak magnetic fields that encode sensory processing, nociception, and cognitive state. Magnetoencephalography (MEG) captures the synchronized firing of pyramidal neurons, producing magnetic fields typically ranging from 50 to 500 fT [23]. Conventional neural monitoring often utilizes microelectrode arrays to reveal neuronal function with high spatial and temporal precision [121]. TMR sensors, capped with a SiO_2_ (50 nm)/Si_3_N_4_ (25 nm)/SiO_2_ (50 nm) layer to ensure biocompatibility and support viable neuron culture, were used for in vitro sensing of neuronal networks in [46]. However, the acquisition of these extracellular fields is fundamentally invasive and poses inherent risks of cellular damage and physiological interference [122]. Optical techniques have emerged as powerful alternatives to overcome these invasive limitations. For instance, dual-objective two-photon microscopy has enabled high-resolution volumetric imaging of dense biological samples [123], while miniaturized systems now allow for large-scale calcium imaging in freely moving subjects [124]. Despite these breakthroughs, optical signals remain an indirect proxy for neural activity—inherently slower than actual electrical transients—and are frequently hindered by phototoxicity and photobleaching during long-term studies [125].

Magnetic sensing circumvents these constraints, offering a non-invasive, high-penetration modality that captures rapid neural pulses at millisecond resolution. By quantifying cortical responses directly, MEG provides an objective, data-driven measure of nociception during routine husbandry procedures, enabling the optimization of analgesia protocols and the establishment of rigorous animal welfare standards [23]. The modularity of OPMs enables wearable, conformal MEG arrays that capture neural signatures of stress and cognition in conscious, moving animals within naturalistic environments [10]. Recently, a brain–computer interface (BCI) based on OPM-MEG has been validated for high-frequency steady-state visual evoked field (SSVEF). The system uses imperceptible ~60 Hz visual flicker stimulation, achieving an offline average accuracy of 92.98% and an information transmission rate of 58.36 bit/min [126].

Beyond central nervous system monitoring, this section also encompasses the electrodynamics of peripheral nerves and skeletal muscles (Magnetomyography, MMG). Although peripheral APs generate significantly weaker fields, resolving nerve conduction and muscle fiber recruitment provides an objective measure of motor control and stress-induced reflexes. These biomagnetic signatures serve as early warning mechanisms for lameness or physical exhaustion, providing a comprehensive framework for autonomic welfare surveillance that bridges central cognitive states with peripheral physiological outcomes.

#### 3.2.3. Digestive Dynamics: Rumen Motility and Gastrointestinal Slow Waves

The digestive efficiency of ruminants is governed by the complex mechanical and electrophysiological coordination of the reticulorumen. In precision livestock farming, magnetic sensing has branched into two primary methodologies: the tracking of artificial magnetic dipoles for mechanical analysis and the detection of endogenous slow waves for metabolic assessment. State-of-the-art magnetic tracking systems developed for wireless capsule robots also provide relevant technological comparisons. Su et al. recently demonstrated a wearable, reconfigurable, and modular magnetic tracking system for capsule robots, highlighting the value of distributed magnetic arrays and adaptable sensor geometries for robust localization in body-mounted settings [127]. Although not designed specifically for ruminants, this architecture offers useful guidance for future rumen bolus tracking systems, particularly in wearable array design and motion-tolerant localization.

The tracking of artificial dipoles is typically achieved via ingestible rumen boluses. These high-density nodes are designed to reside permanently within the reticulum, where they function as moving magnetic sources. External sensor arrays (e.g., fluxgates or MR sensors) positioned on the animal’s flank or within milking stalls can resolve the bolus’s 3D trajectory [128]. The reticulum undergoes a characteristic biphasic contraction cycle to mix ingesta; healthy motility is defined by a consistent contraction frequency of approximately one event per minute [129]. Deviations from this magnetic trajectory serve as high-fidelity diagnostic markers. For instance, a reduction in contraction frequency (hypomotility) is a primary indicator of subclinical metabolic disorders such as milk fever (hypocalcemia) or displaced abomasum [130]. Furthermore, subtle shifts in reticular tone and activity levels, captured through magnetic displacement, have proven effective for estrus detection, aiding in the optimization of insemination timing.

In parallel with mechanical tracking, high-sensitivity magnetometry can resolve the endogenous gastrointestinal (GI) slow waves initiated by the Interstitial Cells of Cajal. These bioelectromagnetic signals, typically residing in the ultra-low frequency range (0.05–0.5 Hz), correlate directly with the intrinsic rhythmicity of nutrient processing. While pH sensors in boluses are frequently used for monitoring Subacute Ruminal Acidosis (SARA), they are notorious for sensor drift in the harsh rumen environment. Magnetic monitoring of GI slow waves offers a more stable alternative, as the rumen wall’s electrical activity becomes suppressed during acidic episodes, providing a reliable proxy for SARA without the need for recalibration [131]. By integrating macroscopic dipole tracking with microscopic slow-wave analysis, magnetic sensing platforms enable a multiscale view of digestive health, supporting precision nutrition strategies that maximize feed conversion efficiency.

### 3.3. Soil–Microbial–Plant Electromagnetic Continuum

Soil is not merely a passive substrate but an active electromagnetic medium shaped by mineralogy, microbial metabolism, and root activity. The magnetic properties of agricultural soils emerge from a combination of abiotic pedogenic processes and biotic drivers, such as microbial respiration and iron cycling. These signals form a functional continuum linking soil chemistry, microbial ecology, and plant physiological state. Magnetometry uniquely captures this integrated behavior, offering a systems-level diagnostic of soil health that transcends traditional chemical assays by providing non-destructive, real-time insights into the subterranean ecosystem. The concept of the soil-microbial-plant electromagnetic continuum is visualized in Figure 3C, showing how LF-MRI and susceptibility mapping resolve subterranean metabolic activities and root architecture.

#### 3.3.1. Soil Magnetic Susceptibility as a Biological Proxy

Soil magnetic susceptibility (χ) reflects the concentration, composition and transformation of ferrimagnetic minerals, primarily magnetite (Fe_3_O_4_) and maghemite (γ-Fe_2_O_3_), which are sensitive to biological modulation. Rather than a purely physical parameter, susceptibility serves as a rapid, non-destructive proxy for soil fertility, redox status, and drainage capacity. In well-drained, oxidative agricultural environments, pedogenic processes favor the accumulation of these minerals, whereas in poorly drained or compacted fields, dissimilatory iron-reducing bacteria use ferric iron (Fe^3+^) as an electron acceptor. Microbial-driven magnetic depletion in hydric soils enables the precise delineation of drainage classes and wetland boundaries, facilitating optimized irrigation and soil management [132].

Beyond natural pedogenesis, soil magnetometry is a robust tool for assessing the biological risks associated with anthropogenic pollution. Industrial and atmospheric technogenic magnetic particles (TMPs) often encapsulate heavy metals such as Pb, Cd, Zn, and Cu [133]. These particles enhance the magnetic susceptibility of topsoil, and field studies have demonstrated strong positive correlations between low-frequency susceptibility ($\chi_{lf}$) and the pollution load index [134]. Utilizing portable fluxgate susceptibility meters allows for the high-density screening of agricultural lands, identifying contamination hotspots that may inhibit microbial health or plant growth without the need for extensive grid sampling and destructive chemical assays [135]. And this method is non-destructive, fast, and significantly cheaper than traditional grid sampling for chemical assay.

#### 3.3.2. Microbial Metabolic Activity and Biogeochemical Signatures

The metabolic vitality of soil microbial communities is a primary driver of nutrient cycling and subterranean health. Electroactive microorganisms, particularly those involved in dissimilatory iron reduction such as *Geobacter* and *Shewanella* species, govern the biogeochemical cycling of iron by utilizing solid-phase ferric oxides as extracellular electron acceptors [136]. This process generates extracellular electron transfer currents that, while individually weak, produce collective magnetic signatures correlated with organic matter mineralization and soil fertility.

By reducing magnetic ferric oxides or transforming them into different mineral phases, these bacteria fundamentally alter soil magnetic susceptibility, a property that has been shown to be highly predictive of bacterial community composition and alpha-diversity [137]. This biomagnetic coupling is particularly significant in specialized agricultural environments such as paddy soils, where iron cycling is intricately linked to carbon sequestration and greenhouse gas emissions [138]. Utilizing high-sensitivity quantum sensors to detect transient metabolic currents and the resulting mineralogical shifts allows for the online assessment of soil metabolic states. This technical pathway facilitates the real-time diagnosis of soil fertility in precision agriculture while avoiding the ecological disturbances associated with traditional destructive sampling and chemical analysis.

#### 3.3.3. Biogenic and Synthetic Magnetic Nanostructures: From Ecological Indicators to Sensing Templates

Biological and engineered magnetic nanostructures provide unique windows into subterranean dynamics and offer versatile platforms for agricultural sensing. This continuum begins with biogenic minerals synthesized by magnetotactic bacteria (MTB), which biomineralize single-domain magnetite (Fe_3_O_4_) or greigite (Fe_3_S_4_) nanocrystals, known as magnetosomes, to navigate geomagnetic fields. In agricultural soils, the spatial distribution and magnetic properties of these nanoparticles encode the redox history and microbial community structure [135]. Monitoring these biogenic particles offers a non-invasive method to evaluate long-term ecosystem stability and iron cycling.

MTB is not only a passive recorder of environmental changes, but also an active participant in agricultural soil ecosystems. Its magnetotaxis enables directional migration along the geomagnetic field to precisely locate at soil redox interfaces [139], thereby coupling iron metabolism with the biogeochemical cycles of carbon, nitrogen, and sulfur. Extracellular polymeric substances secreted by MTB contribute to the formation of stable soil aggregates, improving porosity, water infiltration, and gas exchange to optimize the rhizosphere microenvironment [140]. Upon cell lysis, biogenic iron oxide nanoparticles released from magnetosome chains may modulate root development and stress responses in a manner similar to synthetic iron oxide nanoparticles [141]. Notably, this magnetic behavior originated from adaptive evolution in the early Earth environment, indicating long-term coevolution with the geomagnetic field [142]. Therefore, the vitality of MTB communities serves not only as a record of soil health but as a dynamic agent shaping the below-ground habitat for higher organisms, establishing a direct biological link between the soil’s magnetic properties and agricultural yield.

Complementing these natural indicators, the development of synthetic magnetic nanoporous materials and nanowire arrays has expanded the toolkit for high-precision agricultural diagnostics. Nanoporous templates, such as anodic aluminum oxide (AAO) and zeolites, enable the fabrication of highly ordered magnetic nanostructures with tailored properties. For instance, the synthesis of CoFe_2_O_4_ [143] or Ni nanowire arrays within AAO pores [144] facilitates the creation of high-density magnetic recording media and high-sensitivity sensors. Such nanoconfined environments allow for the precise control of magnetic anisotropy and relaxation behaviors [145], which are critical for developing the next generation of MR biosensors used in detecting food-borne pathogens or soil contaminants [146].

Furthermore, magnetic nanoporous composites serve as transformative functional materials in agricultural chemical engineering. Integrating magnetic clusters or transition metal complexes into the nanocavities of zeolites or mesoporous SiO_2_ creates robust catalysts for the oxidation of organic compounds [147]. A significant advantage of these magnetic nanostructures, such as Au/m-SiO_2_/Fe_3_O_4_ spheres, is their inherent thermal stability and the ability to be separated via external magnetic fields [148]. In the context of precision agriculture, this enables the sustainable recovery and reuse of catalysts in soil remediation or wastewater treatment, significantly reducing operational costs and preventing secondary environmental contamination. By bridging biogenic mineralogy with engineered magnetic nanopores, this multiscale framework enables both the surveillance of natural soil health and the deployment of advanced sensing and remediation technologies.

### 3.4. Aquaculture and Marine Fisheries: Magnetic Sensing in Aquatic Environments

Aquatic environments impose fundamental constraints on optical and radio-frequency sensing due to strong attenuation and scattering in water, rendering magnetic sensing an indispensable modality rather than a complementary option. Building on a long history in geophysical surveying and underwater navigation, recent advances in scalar and vector magnetometers have enabled a transition toward biologically relevant measurements [149]. Deployed on autonomous underwater vehicles, gliders, and towed platforms, modern magnetometry provides robust, spatially resolved observations in optically opaque marine environments where conventional sensing fails [24].

In marine fisheries and aquaculture, this capability directly supports ecological assessment and resource management. Many economically important species rely on geomagnetic cues for orientation and migration [150], while the rapid expansion of offshore infrastructure, including subsea power cables and steel aquaculture enclosures, introduces localized magnetic perturbations that may have minimal behavioral effects [151]. At the same time, inductive and geomagnetic tagging technologies enable non-invasive, long-term tracking of individuals across both farmed and open-ocean systems [152]. Together, these applications position magnetic sensing as a physically grounded, field-deployable tool for precision tracking, behavioral monitoring, and sustainable fisheries management.

#### 3.4.1. Precision Tracking via Inductive and Geomagnetic Tagging

Individual identification and inventory management are critical for optimizing feed conversion ratios and health protocols in high-density aquaculture. Passive Integrated Transponders (PIT) serve as the industry standard, utilizing inductive coupling to energize encapsulated copper coils that transmit unique identification codes when fish pass through magnetic loop antennas [153,154]. This battery-free modality provides a robust, lifetime tracking solution for granular monitoring of growth rates and vaccination history. For open-water applications such as sea ranching, magnetic archival tags reconstruct migration routes by logging spatiotemporal variations in geomagnetic intensity and inclination [155,156]. This geolocation methodology is essential for the stock assessment of high-value migratory species including tuna and salmon.

#### 3.4.2. Geomagnetic Navigation and Anthropogenic Interference

Many economically significant aquatic species, including salmonids and anguillid eels, possess well-documented magnetoreceptive capabilities that support migration, orientation, and habitat selection behaviors [157,158,159]. Because these behaviors are closely linked to physiological adaptation and environmental sensing, magnetic perception provides an important target for biosensing in aquatic systems. Anthropogenic magnetic disturbances generated by subsea infrastructure and aquaculture facilities can perturb local geomagnetic conditions within the sensitivity range of magnetoreceptive organisms, potentially altering swimming behavior, migration timing, and stress responses [151,160,161]. In this context, precision magnetometry enables non-invasive monitoring of biologically relevant behavioral responses to magnetic-field perturbations. By correlating spatial magnetic anomalies with movement trajectories and physiological indicators, magnetic sensing platforms can support stress assessment, welfare monitoring, and adaptive aquaculture management [160,162].

In addition, magnetic guidance systems exploiting species-specific magnetosensory responses have been explored as low-stress alternatives to physical containment strategies [163,164,165,166,167]. Coupled with physiological monitoring, such approaches may contribute to closed-loop biosensing frameworks for intelligent aquatic farming systems.

### 3.5. Developmental and Collective Bioelectromagnetic Dynamics

Beyond organ-level signals, agricultural systems exhibit bioelectromagnetic patterns that emerge over developmental timescales and across interacting individuals. Persistent ionic fluxes associated with tissue growth, vascular differentiation, and organ maturation generate slow, spatially organized magnetic fields that precede visible morphological change [168,169]. As these fields integrate physiological activity over extended time windows, they provide early, non-invasive indicators of developmental status and growth stability under field-relevant conditions.

At the population scale, bioelectromagnetic activity increasingly reflects collective physiological coupling rather than isolated organ function. In plant communities, shared rhizosphere and hydraulic networks enable coordinated electrical responses to environmental stress [93], while in livestock, synchronized cardiac and neural dynamics emerge during collective behavior or common perturbations [170,171]. Coherent temporal structure in magnetic recordings across individuals thus offers a quantitative measure of population-level synchrony, with deviations indicating emerging stress, disease, or environmental mismatch [172]. SERF OPM has successfully demonstrated group measurement capability, enabling rapid, accurate quantification of magnetic nanoparticle concentrations (down to 0.1 mg/mL in 2 μL samples, <11.8 ng error) and flow rates for minimal-sample biomedical applications like immunoassays [173].

From a sensing perspective, developmental and collective bioelectromagnetic dynamics occupy an intermediate regime between localized electrophysiological signals and ecosystem-scale indicators. Their spatially distributed and temporally integrated nature renders them robust to local variability and suitable for continuous, non-invasive monitoring [174]. When combined with organ-resolved and soil-associated magnetic observables, these signals complete a multiscale sensing framework that links individual physiology, population behavior, and environmental context, enabling anticipatory management rather than reactive intervention.

### 3.6. Field-Compatible Biomagnetic Biosensing

A major challenge in agricultural magnetic biosensing is the detection of ultra-weak biomagnetic signals under unshielded environmental conditions. Early biomagnetic measurements relied heavily on Magnetically Shielded Rooms (MSRs) to suppress the Earth’s magnetic field (~50 µT) and urban noise, severely limiting deployment outside laboratory environments. Recent advances in OPM gradiometry, active noise cancelation, and low-noise shielding have significantly improved the feasibility of field-compatible biomagnetic sensing [175,176]. In practical biosensing configurations, gradiometric measurement suppresses spatially correlated environmental noise while preserving localized physiological biomagnetic signals such as cardiac magnetic activity [177]. Recent studies have successfully recorded MCG signals from cattle in unshielded barn environments using OPM arrays [69], thereby demonstrating the viability of on-site cardiac monitoring in non-laboratory, real-world settings. Active compensation coils can further reduce geomagnetic interference and improve signal stability during long-term physiological monitoring [91,178,179,180].

In parallel, passive low-noise magnetic shielding based on high permeability materials remains important for improving signal-to-noise ratio and sensitivity in weak-field biosensing applications [65,181,182,183,184,185]. Such strategies are particularly relevant for low-amplitude measurements including fMCG [119], and low-field magnetic resonance detection.

Together, these advances are accelerating the translation of biomagnetic sensing from laboratory instrumentation toward practical biosensor platforms for continuous plant and animal health monitoring in agricultural environments. This trend provides a feasible pathway for on-site plant electrophysiology and livestock cardiac monitoring in precision agriculture, significantly expanding the practical potential of magnetic biosensing in real-world agriculture settings.

## 4. The Role of AI and DTs

**Figure 4:**
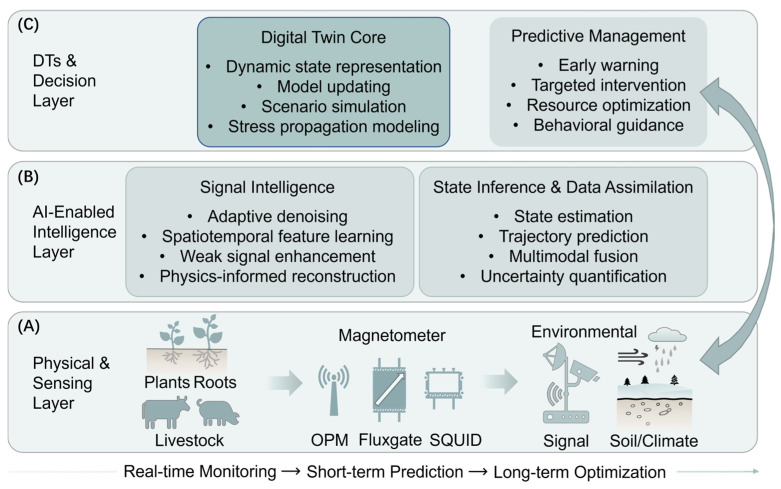
Hierarchical architecture of an AI-enabled Digital Twin (DT) framework for precision agricultural magnetometry. The diagram illustrates a multi-tiered system for translating raw magnetic signals into actionable agricultural intelligence through a closed-loop feedback mechanism. (**A**) Physical and Sensing Layer: The foundation involves multi-modal data acquisition from biological (e.g., livestock electrophysiology, root phenotyping) and environmental (e.g., soil magnetic susceptibility, climatic flux) sources using high-sensitivity magnetometers (OPM, Fluxgate, and SQUIDs). (**B**) AI-Enabled Intelligence Layer: This intermediate stage bridges raw data and high-level modeling. It integrates Signal Intelligence—utilizing adaptive denoising and physics-informed reconstruction to resolve weak bio-magnetic signals—with State Inference and Data Assimilation to quantify uncertainty and fuse multi-source information. (**C**) DTs and Decision Layer: The apex of the architecture comprises the Digital Twin Core, which manages dynamic state representation and stress propagation modeling. This enables Predictive Management, facilitating targeted interventions, resource optimization, and behavioral guidance. The iterative process flows from real-time monitoring to short-term prediction and culminates in long-term system optimization, ensuring a robust synergy between physical sensing and digital representation.

The deployment of high-sensitivity magnetic sensors in agricultural environments fundamentally transforms bioelectromagnetic observation into a data-intensive problem. Despite their immense potential to revolutionize next-generation information systems through integration with future wireless systems, quantum-enabling technologies in communication, computing, and sensing still face fundamental challenges including environmental noise, limited entanglement coherence times, and a lack of specialized quantum devices [186]. Continuous recordings from OPM arrays operating in unshielded barns, greenhouses, or open fields are dominated by nonstationary environmental noise, platform-induced interference, and physiological variability [187]. Similarly, LF-MRI of soils or root systems produces sparse, low-SNR measurements that are difficult to interpret using conventional analytical pipelines. These challenges are increasingly mitigated through the deployment of advanced algorithms and computational models [188]. In this context, AI functions not as a replacement for physical modeling, but as an interpreter that maps high-dimensional, noisy sensor outputs onto biologically and agronomically meaningful representations. As synthesized in Figure 4, this integration is structured as a hierarchical framework that bridges the physical sensing layer with an AI-driven intelligence layer, ultimately informing a DT decision layer for predictive management.

The physical sensing layer may also include magnet-related sensors that monitor mechanical interactions rather than endogenous biomagnetic fields. For instance, Zhang et al. demonstrated a magnetoelastic torque sensor with planar spiral coil probes for humanoid robot joints, providing an example of magnetic transduction for mechanical-state monitoring [189]. Although outside direct agricultural biosignal detection, such designs are relevant to agricultural robotics, wearable devices, and livestock-handling systems, where torque, contact force, and motion variables can complement physiological magnetic signals in AI-enabled DTs.

### 4.1. Deep Learning for Signal Extraction and Noise Suppression

In agricultural biosensing, target signals—such as sub-pT magnetic transients from plant action potentials or fT-level neural activity—are buried in backgrounds orders of magnitude stronger, including Earth’s ~50 µT field, infrastructure interference, and motion artifacts. Conventional linear filters and hardware gradiometry falter in nonstationary, mobile, or unshielded scenarios.

Deep learning advances offer data-driven solutions by leveraging spatial-temporal correlations in sensor arrays. Convolutional neural networks (CNNs) and recurrent models like long short-term memory (LSTMs) train on paired noise-dominated reference and signal-plus-noise primary channels to map nonlinear interference patterns [190]. This yields synthetic gradiometers surpassing traditional adaptive filtering for noise cancelation. Demonstrated in magnetoencephalography and OPM recordings, AI-assisted denoising enhances signal detection in unshielded settings, facilitating field-deployable biosensing [191]. Deep denoising autoencoders and CNN-based filters enhance distributed strain sensing in crop monitoring, clarifying bioelectric signals under environmental variability [192].

For LF-MRI, k-space data suffer from undersampling and noise at ultra-low fields due to weak thermal polarization. The AUTOMAP framework employs neural networks to map sensor-domain data directly to image space, reconstructing accurate images from noisy, sparse inputs—originally for medical MRI but adaptable to agriculture for root and soil imaging [109]. In precision agriculture, these techniques integrate with IoT sensors for crop health monitoring, optimizing irrigation and pest management through enhanced electromagnetic and acoustic data analysis [193].

### 4.2. Foundation Models for Multimodal Agro-Biosignal Integration

Pre-training techniques, a cornerstone of deep learning, involve initial training on large-scale datasets to capture general features before fine-tuning on task-specific data, addressing data scarcity and high annotation costs [194]. This enhances generalization and performance in complex tasks like protein structure prediction and drug design [195,196]. Large-scale models trained on gene expression data handle biomedical tasks and adapt to diverse sources [197]. Multimodal models like BioCLIP integrate images and structured knowledge for biological queries [198]. For noise suppression, pre-training captures complex noise patterns and signal essences, enabling effective interference removal even with limited labeled data [199]. Architectures like CNNs or RNNs identify and separate noise types, improving adaptability to nonstationary conditions and enabling real-time applications in agricultural settings [200].

Extending to agricultural breeding, pre-trained AI models revolutionize gene and protein prediction by integrating genomic, proteomic, and bioelectromagnetic data. Tools like AlphaFold and ESMFold predict protein structures with high accuracy, enabling the identification of functional domains in crop genes for traits such as disease resistance and yield improvement [201]. In maize, benchmarking these models on key genes highlights their role in decoding protein folding, aiding breeding for resilient varieties [202]. AI-driven genomic prediction, such as SoyDNGP, optimizes parent selection and multi-trait integration, accelerating crop innovation by modeling complex interactions like epistasis [203]. WheatGP, combining CNN and LSTM, captures additive and epistatic effects for accurate phenotype prediction in wheat traits [204]. In the field of poultry disease resistance, based on gene editing tools, rapid detection of α-herpesvirus can be achieved [205]. By fusing magnetic sensor data (e.g., plant stress signals) with omics datasets, these models predict gene expression and regulatory elements, supporting precision breeding for enhanced sustainability and productivity [206]. Emerging genomic language models like AgroNT, pre-trained on crop DNA, enable tasks such as variant effect prediction and promoter identification, further advancing orphan crop improvement [207].

Ultimately, foundation models leverage multimodal alignment to synchronize dynamic magnetic signatures with static omic profiles, bridging the inherent gap between real-time physiology and genetic architecture. This synergy establishes the algorithmic groundwork for agricultural scientific foundation models, shifting the paradigm from reactive monitoring toward the proactive, multiscale design of resilient biological systems.

### 4.3. DTs as Predictive Integrators of Magnetic and Physiological Data

The ultimate value of AI-enabled magnetic sensing emerges when signal extraction is coupled to predictive, system-level models. DTs provide such a framework by linking real-time sensor data to dynamic computational representations of biological assets. In agriculture, DTs are increasingly applied not only to physical infrastructure, but also to living organisms, incorporating physiological state variables into continuously updated models.

In livestock systems, DTs can integrate heterogeneous magnetic observables—such as rumen motility inferred from magnetically tracked boluses, cardiac dynamics derived from magnetocardiography, and neural or muscular activity captured via wearable OPMs—with environmental and behavioral data streams. By ingesting real-time streams from rumen boluses (motility), OPM collars (heart rate/variability), and environmental sensors, a DT can model the individual animal’s metabolic and health trajectory [208].

For example, a dairy-cow ketosis DT could be organized as a four-stage workflow. First, the input layer would acquire time-series magnetic field measurements from rumen capsule tracking, from which bolus displacement, three-dimensional trajectory, contraction frequency, contraction amplitude, and inter-contraction intervals are derived. Simultaneously, OPM-based magnetocardiography would provide cardiac magnetic waveforms, heart rate, heart-rate variability, and rhythm-irregularity indices, supplemented by non-magnetic variables such as feed intake, milk yield, activity level, body temperature, and barn temperature or humidity. Second, the feature-fusion layer would align these heterogeneous streams on a common temporal axis and remove motion or environmental artifacts using adaptive filtering or learning-based denoising. Third, the predictive layer could combine recurrent neural networks, such as LSTM models, with physiological state-space models to infer latent variables related to energy balance, rumen function, and metabolic stress. Finally, the output layer would generate individualized ketosis-risk scores, early-warning alerts, and interpretable physiological indicators, which could be validated against blood beta-hydroxybutyrate concentration, milk ketone tests, clinical diagnosis, milk-yield changes, and longitudinal herd-health records.

Such a framework would allow the DT to detect a subtle drift in the magnetic rumen signature combined with declining heart-rate variability, thereby predicting a metabolic disorder before clinical signs become apparent. This allows the farmer to intervene proactively—adjusting feed or administering supplements—moving management from reactive treatment to predictive prevention [209].

From a broader perspective, the integration of AI-driven signal extraction with DT frameworks closes the loop between sensing and decision-making. Magnetic biosignals, which are inherently non-invasive and continuously observable, are particularly well suited for this paradigm. When embedded within DTs, they enable anticipatory management strategies that operate on physiological trajectories rather than threshold-based alarms, aligning precision agriculture with principles of resilience, welfare, and sustainability.

## 5. Conclusions and Prospects

Despite the expanding range of demonstrated applications across soil–plant systems, livestock monitoring, aquaculture, and AI-enabled data integration, the practical deployment of magnetic biosensing in agriculture remains constrained by several technical and translational challenges. Chief among these is reliable operation in unshielded, dynamic environments, where ultra-sensitive magnetic biosensor readout platforms must resolve pT to fT biological signals against the ~50 µT geomagnetic background and time-varying anthropogenic interference. While advances in active field cancelation, gradiometric configurations, and AI-based synthetic gradiometry have significantly relaxed shielding requirements, robust performance on moving platforms, such as free-ranging animals, mobile field robots, or offshore installations, remains an open engineering challenge. Additional limitations arise from bandwidth–sensitivity trade-offs across biological timescales, sensor cost and scalability, long-term calibration stability, and lack of standardized protocols linking magnetic observables to actionable agricultural metrics.

From a biosensor perspective, the central significance of agricultural magnetometry lies in its ability to extend biosensing beyond surface-accessible optical or chemically mediated readouts. Magnetic approaches can operate as label-free transduction platforms for endogenous biological activity, including ionic currents, cardiac and gastrointestinal electrophysiology, plant action potentials, and magnetically mediated soil or microbial processes. In parallel, magnetic labels, biofunctional materials, magnetoelastic elements, and molecular-recognition strategies provide complementary routes for coupling magnetic readout with more conventional biosensor recognition mechanisms. Thus, the field should not be viewed simply as the application of physical magnetometers to agriculture, but as an emerging magnetic biosensing framework in which biological information is converted into measurable magnetic signatures.

Looking ahead, the value of magnetic biosensing in agriculture will increasingly depend on its integration into multiscale, predictive sensing frameworks rather than on isolated measurements. Magnetic biosignals uniquely span spatial and temporal domains, connecting subcellular ionic currents and organ-level electrophysiology with population-scale coordination and environmental interactions. The convergence of magnetic biosensing with AI-driven signal processing and digital twin methodologies represents a critical step toward this goal. Within such frameworks, magnetic observables can serve as dynamically updated biomarkers that support data-informed agricultural management and precision intervention strategies.

In conclusion, magnetic biosensing offers a distinct and complementary sensing modality for agricultural and animal systems, providing non-invasive access to physiological processes that are poorly captured by optical, chemical, or electrical methods, particularly in opaque and heterogeneous environments. As this field evolves from classical magnetic sensors toward quantum-enabled biosensing platforms, the central challenge shifts from signal detectability toward reliable biological integration, deployment robustness, and scalable biosensing integration. Continued progress will rely on the co-development of sensor hardware, data-driven analytics, and application-specific validation frameworks. When aligned with the practical constraints of agricultural deployment, magnetic biosensing has the potential to become a robust component of next-generation biosensor technologies for precision and sustainable agriculture.

## Figures and Tables

**Table 1:** Magnetometer technologies and suitability across domains.

Sensor Type	Typical Sensitivity	Bandwidth	Ruggedness	Potential Agro-Use
Fluxgate	0.01–1 nT/Hz^1/2^	DC–3 kHz	High	Soil mapping, Drainage pipe detection [89]
Hall Effect	10–10,000 nT/Hz^1/2^	DC–100 kHz	High	Machinery position, Grain flow monitoring [48]
GMR/TMR	0.1–10 nT/Hz^1/2^	DC–MHz	High	Bolus tracking, Arrays [66]
LF NMR	~1000 nT/Hz^1/2^	Hz–kHz	Moderate	Soil water [22,86], Root imaging [90]
NV Center	0.01–1 nT/Hz^1/2^	DC–GHz	High	Micro-sensing, Tracers [52]
SERF OPMs	1–10 fT/Hz^1/2^	DC–100 Hz	Low (Temp/Field sensitive)	Livestock MCG, Plant Electrophysiology [23]
SQUIDs	0.1–1 fT/Hz^1/2^	DC–GHz	Poor (Cryogenic)	Lab Reference, fetal magnetocardiography (fMCG) [11]

**Table 2:** Typical amplitudes and frequency ranges of biomagnetic signals relevant to agriculture, contrasted with the geomagnetic background.

Biological Signal Source	Typical Sensitivity	Bandwidth	Ruggedness	Potential Agro-Use
Human Brain (MEG)	50–500 fT	0.1–100 Hz	Cortical currents	Extreme sensitivity required
Plant Action Potential	0.5–1 pT	0.1–10 Hz	Ion channels (Cl^−^, Ca^2+^)	Very slow, ultra-weak signal
Fetal Heart (Cow)	1–10 pT	1–50 Hz	Cardiomyocytes	Separating from maternal signal
Adult Heart (Cow)	10–100 pT	1–100 Hz	Cardiomyocytes	Environmental noise overlap
Soil Magnetic Susceptibility	High (induced)	Static/LF	Magnetite/Maghemite	Differentiating anthropogenic vs. pedogenic
Geomagnetic Background	~50,000,000 pT (50 µT)	DC	Earth’s Core	Must be subtracted (6 orders of magnitude larger)

## Data Availability

The data analyzed during the study are available from the corresponding author upon reasonable request.
